# Supplementary material for: The translation and validation of the Organ Transplant Symptom and Well-Being Instrument in China
Source: PLOS Glob Public Health. 2022 Sep 28;2(9):e0000718. doi: 10.1371/journal.pgph.0000718 (PMC10021454; doi:10.1371/journal.pgph.0000718)
Supplement: S1 Text — (DOCX) [file pgph.0000718.s001.docx]

| 器官移植患者健康状况量表 | | | | | | |  |
| --- | --- | --- | --- | --- | --- | --- | --- |
| 下面是和您有同样健康状况的人们列出的一份认为重要的一些陈述。请在以下每一行陈述中标出您认为最合适的表达，用以表明以下每条陈述与您过去7天内的个人情况的符合程度。 | | | | | | |  |
|  |  |  |  |  |  |  |  |
| 1 | 我入睡困难 | 完全不符合 | 几乎不符合 | 有点符合 | 比较符合 | 完全符合 |  |
| 2 | 我睡眠质量不佳 | 完全不符合 | 几乎不符合 | 有点符合 | 比较符合 | 完全符合 |  |
| 3 | 我会半夜醒来 | 完全不符合 | 几乎不符合 | 有点符合 | 比较符合 | 完全符合 |  |
| 4 | 我肌肉疼痛 | 完全不符合 | 几乎不符合 | 有点符合 | 比较符合 | 完全符合 |  |
| 5 | 我关节疼痛 | 完全不符合 | 几乎不符合 | 有点符合 | 比较符合 | 完全符合 |  |
| 6 | 我腿疼 | 完全不符合 | 几乎不符合 | 有点符合 | 比较符合 | 完全符合 |  |
| 7 | 我的脚有烧灼感 | 完全不符合 | 几乎不符合 | 有点符合 | 比较符合 | 完全符合 |  |
| 8 | 我的下肢和脚有麻木和刺痛的感觉 | 完全不符合 | 几乎不符合 | 有点符合 | 比较符合 | 完全符合 |  |
| 9 | 我感到身体疲惫 | 完全不符合 | 几乎不符合 | 有点符合 | 比较符合 | 完全符合 |  |
| 10 | 我感觉没有精力 | 完全不符合 | 几乎不符合 | 有点符合 | 比较符合 | 完全符合 |  |
| 11 | 我感觉倦怠、无精打采 | 完全不符合 | 几乎不符合 | 有点符合 | 比较符合 | 完全符合 |  |
| 12 | 我感觉记不住事 | 完全不符合 | 几乎不符合 | 有点符合 | 比较符合 | 完全符合 |  |
| 13 | 我很难集中精力 | 完全不符合 | 几乎不符合 | 有点符合 | 比较符合 | 完全符合 |  |
| 14 | 由于身体状况，我不能淋浴或泡澡 | 完全不符合 | 几乎不符合 | 有点符合 | 比较符合 | 完全符合 |  |
| 15 | 由于身体原因，我不能自己穿衣服 | 完全不符合 | 几乎不符合 | 有点符合 | 比较符合 | 完全符合 |  |
| 16 | 由于身体原因，我不能自己外出购物 | 完全不符合 | 几乎不符合 | 有点符合 | 比较符合 | 完全符合 |  |
| 17 | 我感到烦躁 | 完全不符合 | 几乎不符合 | 有点符合 | 比较符合 | 完全符合 |  |
| 18 | 我感到气愤 | 完全不符合 | 几乎不符合 | 有点符合 | 比较符合 | 完全符合 |  |
| 19 | 由于我的健康状况，我担心自己无法保住工作 | 完全不符合 | 几乎不符合 | 有点符合 | 比较符合 | 完全符合 |  |
| 20 | 由于我的健康状况，我担心自己的经济状况 | 完全不符合 | 几乎不符合 | 有点符合 | 比较符合 | 完全符合 |  |
| 21 | 我觉得喘不上气 | 完全不符合 | 几乎不符合 | 有点符合 | 比较符合 | 完全符合 |  |
| 22 | 我因喘不过气来需要休息 | 完全不符合 | 几乎不符合 | 有点符合 | 比较符合 | 完全符合 |  |
| 23 | 我有浮肿 | 完全不符合 | 几乎不符合 | 有点符合 | 比较符合 | 完全符合 |  |
| 24 | 我感觉恶心 | 完全不符合 | 几乎不符合 | 有点符合 | 比较符合 | 完全符合 |  |
| 25 | 我有口腔真菌感染 | 完全不符合 | 几乎不符合 | 有点符合 | 比较符合 | 完全符合 |  |
| 26 | 我有口腔疱疹 | 完全不符合 | 几乎不符合 | 有点符合 | 比较符合 | 完全符合 |  |
| 27 | 我食欲增加了 | 完全不符合 | 几乎不符合 | 有点符合 | 比较符合 | 完全符合 |  |
| 28 | 我食欲减退了 | 完全不符合 | 几乎不符合 | 有点符合 | 比较符合 | 完全符合 |  |
| 29 | 我消化不良 | 完全不符合 | 几乎不符合 | 有点符合 | 比较符合 | 完全符合 |  |
| 30 | 我便秘 | 完全不符合 | 几乎不符合 | 有点符合 | 比较符合 | 完全符合 |  |
| 31 | 我腹泻 | 完全不符合 | 几乎不符合 | 有点符合 | 比较符合 | 完全符合 |  |
| 32 | 我皮肤瘙痒 | 完全不符合 | 几乎不符合 | 有点符合 | 比较符合 | 完全符合 |  |
| 33 | 我头疼 | 完全不符合 | 几乎不符合 | 有点符合 | 比较符合 | 完全符合 |  |
| 34 | 我的手有烧灼感 | 完全不符合 | 几乎不符合 | 有点符合 | 比较符合 | 完全符合 |  |
| 35 | 我的手有麻木和刺痛的感觉 | 完全不符合 | 几乎不符合 | 有点符合 | 比较符合 | 完全符合 |  |
| 36 | 我手抖 | 完全不符合 | 几乎不符合 | 有点符合 | 比较符合 | 完全符合 |  |
| 37 | 我感觉头晕 | 完全不符合 | 几乎不符合 | 有点符合 | 比较符合 | 完全符合 |  |
| 38 | 我觉得难过 | 完全不符合 | 几乎不符合 | 有点符合 | 比较符合 | 完全符合 |  |
| 39 | 我的样子让我觉得尴尬 | 完全不符合 | 几乎不符合 | 有点符合 | 比较符合 | 完全符合 |  |
| 40 | 我的性欲下降了 | 完全不符合 | 几乎不符合 | 有点符合 | 比较符合 | 完全符合 |  |
