# Supplementary material for: The translation and validation of the Organ Transplant Symptom and Well-Being Instrument in China
Source: PLOS Glob Public Health. 2022 Sep 28;2(9):e0000718. doi: 10.1371/journal.pgph.0000718 (PMC10021454; doi:10.1371/journal.pgph.0000718)
Supplement: S2 Text — (DOCX) [file pgph.0000718.s002.docx]

| The Organ Transplant Symptom and Wellbeing Instrument (OTSWI) | **0** | **1** | **2** | **3** | **4** |
| --- | --- | --- | --- | --- | --- |
| Below there is a list of statements that other persons, with the same health condition as you, consider important. Please state how well each statement reflects your situation during the last seven days.Please mark the most suitable figure at each line. |  |  |  |  |  |
| I have difficulties with falling asleep |  |  |  |  |  |
| I sleep poorly |  |  |  |  |  |
| I wake up during the night |  |  |  |  |  |
| My muscles are aching |  |  |  |  |  |
| My joints are aching |  |  |  |  |  |
| My legs are aching |  |  |  |  |  |
| There is a burning ache in my feet |  |  |  |  |  |
| There is a numb and stabbing feeling in my feet |  |  |  |  |  |
| I am physically tired |  |  |  |  |  |
| I have no energy |  |  |  |  |  |
| I feel lazy and listless |  |  |  |  |  |
| I have difficulties to remember |  |  |  |  |  |
| I find it hard to concentrate |  |  |  |  |  |
| Due to my physical condition I can’t take a bath or shower |  |  |  |  |  |
| Due to my physical condition I can’t get dressed by myself |  |  |  |  |  |
| Due to my physical condition I can’t buy food by myself |  |  |  |  |  |
| I feel irritated |  |  |  |  |  |
| I feel angry |  |  |  |  |  |
| I worry about not being able to keep my job due to my health condition |  |  |  |  |  |
| I worry about my economy due to my health condition |  |  |  |  |  |
| I’m breathless |  |  |  |  |  |
| I need to rest because I am breathless |  |  |  |  |  |
| I’m swollen |  |  |  |  |  |
| I feel nauseous |  |  |  |  |  |
| I have oral fungus |  |  |  |  |  |
| I have oral herpes |  |  |  |  |  |
| I have increased appetite for food |  |  |  |  |  |
| I have decreased appetite for food |  |  |  |  |  |
| I have dyspepsia |  |  |  |  |  |
| I’m constipated |  |  |  |  |  |
| I have diarrhea |  |  |  |  |  |
| My skin is itching |  |  |  |  |  |
| I have headache |  |  |  |  |  |
| There is a burning pain in my hands |  |  |  |  |  |
| There is a numb and stabbing pain in my hands |  |  |  |  |  |
| My hands are trembling |  |  |  |  |  |
| I feel dizzy |  |  |  |  |  |
| I feel sad |  |  |  |  |  |
| My looks makes me embarrassed |  |  |  |  |  |
| My libido is decreased |  |  |  |  |  |
