# Supplementary material for: The translation and validation of the Organ Transplant Symptom and Well-Being Instrument in China
Source: PLOS Glob Public Health. 2022 Sep 28;2(9):e0000718. doi: 10.1371/journal.pgph.0000718 (PMC10021454; doi:10.1371/journal.pgph.0000718)
Supplement: Attachment — Submitted filename: Response to Reviewers.docx [file pgph.0000718.s003.docx]

Dear Editors and Reviewers:

Thank you for your letter and for the reviewers’ comments concerning our manuscript entitled " The translation and validation of the Organ Transplant Symptom and Well-Being instrument in China " ( PGPH-D-21-00455). Those comments are all valuable and very helpful for revising and improving our paper, as well as the important guiding significance to our research. We have studied comments carefully and have made correction which we hope to meet with approval. Revised portions are marked in red in the paper. The main corrections in the paper and the responds to the reviewer’s comments are as flowing:

Dear Dr. LI,

Thank you for submitting your manuscript to PLOS Global Public Health. After careful consideration, we feel that it has merit but does not fully meet PLOS Global Public Health’s publication criteria as it currently stands. Therefore, we invite you to submit a revised version of the manuscript that addresses the points raised during the review process.

Please submit your revised manuscript by Jun 09 2022 11:59PM. If you will need more time than this to complete your revisions, please reply to this message or contact the journal office at globalpubhealth@plos.org. When you're ready to submit your revision, log on to https://www.editorialmanager.com/pgph/ and select the 'Submissions Needing Revision' folder to locate your manuscript file.

Please include the following items when submitting your revised manuscript:

- A rebuttal letter that responds to each point raised by the editor and reviewer(s). You should upload this letter as a separate file labeled 'Response to Reviewers'.
- A marked-up copy of your manuscript that highlights changes made to the original version. You should upload this as a separate file labeled 'Revised Manuscript with Track Changes'.
- An unmarked version of your revised paper without tracked changes. You should upload this as a separate file labeled 'Manuscript'.

Guidelines for resubmitting your figure files are available below the reviewer comments at the end of this letter.

We look forward to receiving your revised manuscript.

Kind regards,

Javier H Eslava-Schmalbach, M.D., Ph.D., MSc
Academic Editor
PLOS Global Public Health

Journal Requirements:

1. Please send a completed 'Competing Interests' statement, including any COIs declared by your co-authors. If you have no competing interests to declare, please state "The authors have declared that no competing interests exist".

Answer: Thank you for your question, "The authors have declared that no competing interests exist"

2. Please amend your detailed Financial Disclosure statement. This is published with the article. It must therefore be completed in full sentences and contain the exact wording you wish to be published.

If you did not receive any funding for this study, please simply state: “The authors received no specific funding for this work.”

Answer: Thank you for your question, "The authors received no specific funding for this work.”

3. We do not publish any copyright or trademark symbols that usually accompany proprietary names, eg (R), (C), or TM  (e.g. next to drug or reagent names). Please remove all instances of trademark/copyright symbols throughout the text, including (C) on page 21.

Answer: Thank you for your question, I have revised your question and removed the problem that existed on page 21

4. In the online submission form you indicate that your data is not available for proprietary reasons and have provided a contact point for accessing this data. Please note that your current contact point is a co-author on this manuscript. According to our Data Policy, the contact point must not be an author on the manuscript and must be a third party. Please revise your data statement to a non-author institutional point of contact, such as a data access or ethics committee, and send this to us via return email. Please also include contact information for the third party organization, and please include the full citation of where the data can be found.

Additional Editor Comments (if provided):

Dear author:

We have finally received comments from our reviewers: Please respond/include each one of their observations. Also, please respond/include these suggestions:

1. Check the correct use of English in the manuscript, so the adequate use of terms like: farm, retire, man, woman

2. The test-retest reliability section should be corrected. You suggest to use one test, and finally ended up using other.

3. References: 3, 13, 53, should be double-checked

4. Include texts in supplementary material, in both languages


[Note: HTML markup is below. Please do not edit.]

Reviewers' comments:

Reviewer's Responses to Questions

**Comments to the Author**

1. Does this manuscript meet PLOS Global Public Health’s [publication criteria](https://journals.plos.org/globalpublichealth/s/criteria-for-publication)? Is the manuscript technically sound, and do the data support the conclusions? The manuscript must describe methodologically and ethically rigorous research with conclusions that are appropriately drawn based on the data presented.

Reviewer #1: Yes

Reviewer #2: Yes

2. Has the statistical analysis been performed appropriately and rigorously?

Reviewer #1: No

Reviewer #2: Yes

3. Have the authors made all data underlying the findings in their manuscript fully available (please refer to the Data Availability Statement at the start of the manuscript PDF file)?

The [PLOS Data policy](https://journals.plos.org/globalpublichealth/s/data-availability) requires authors to make all data underlying the findings described in their manuscript fully available without restriction, with rare exception. The data should be provided as part of the manuscript or its supporting information, or deposited to a public repository. For example, in addition to summary statistics, the data points behind means, medians and variance measures should be available. If there are restrictions on publicly sharing data—e.g. participant privacy or use of data from a third party—those must be specified.

Reviewer #1: Yes

Reviewer #2: Yes

4. Is the manuscript presented in an intelligible fashion and written in standard English?

PLOS Global Public Health does not copyedit accepted manuscripts, so the language in submitted articles must be clear, correct, and unambiguous. Any typographical or grammatical errors should be corrected at revision, so please note any specific errors here.

Reviewer #1: Yes

Reviewer #2: Yes

5. Review Comments to the Author

Please use the space provided to explain your answers to the questions above. You may also include additional comments for the author, including concerns about dual publication, research ethics, or publication ethics. (Please upload your review as an attachment if it exceeds 20,000 characters)

Reviewer #1: All comments and suggestions are in the attached files. The quality of the study was evaluated applying the STROBE checklist and the recommendations by section were abandoned.


1. It is considered important that the authors include a paragraph on the control of bias in the study. **Answer: Thank you for your question, I have revised your question and removed the problem that existed on page 21**

2. The study proposes a factorial analysis. Given that there is a hypothesis about the factorial structure of the instrument, it is suggested being explicit about that hypothesis and indicate that it is NOT an exploratory analysis, but a confirmatory one; and make explicit why it was decided to do a factorial analysis of principal components using a varimax rotation.
Finally, ¿why does the study use the Pearson correlation and not the Spearman correlation if the variables are ordinal? I suggest change that correlation or show that the variables meet the assumptions.

**Answer: Thank you for your question. The analysis used in the article was conducted by Spearman, and it was our lack of rigor that led to the writing error.**

3. In the methodology the authors say that “A value of > 0.40 was considered to be substantial” and then in the results the authors say that “A correlation coefficient of lower than 0.3 was adopted as the basis for question deletion”
Why they change the first assumption? A correlation of 0.3 is considered low, and a candidate for deletion.
The authors say “Correlations between individual items and the total score of the C-OTSWI were significant and positive for the entire sample.”
But It’s not the same the statistical significance and the strength of association. Seven items have a correlation between individual items lower that 0.4 and two lower that 0.3; It’s important analyse is it’s better eliminate those items or explain very well why they don’t do that.
In the methodology the authors say that they will use a Pearson correlation, but in the results, they write about a Spearman’s rank correlation coefficient and then report an intraclass correlation coefficients as a result. They must change that and be consistent between methodology and results.

4. The authors include some limitations, but nothing about the lack of bias control

5. The authors say: “The high correlation coefficient shows that the items of the scale have a strong relationship to the scale construct.”
There was not high correlation in all cases, my recommendation is to be conservative in the discussion and more precise based on the results

Reviewer #2: The manuscript responds to a need in evaluating the outcomes and prognosis of transplant activity. The use of the OTSWI instrument has broadened the horizon of the evaluation of transplant patients beyond physical well-being and allows the senses of physical, mental and emotional well-being to be included. The methodology used both to adapt the scale from the point of view of the language and with the linguistic and cultural aspects of the conception of the disease, is absolutely appropriate. Regarding the methodology to evaluate the validity and internal consistency, reliability and concurrent validity, the approach, the results and the significance are interesting and clearly important and applicable.

6. PLOS authors have the option to publish the peer review history of their article ([what does this mean?](https://journals.plos.org/globalpublichealth/s/editorial-and-peer-review-process#loc-peer-review-history)). If published, this will include your full peer review and any attached files.


**Do you want your identity to be public for this peer review?** If you choose “no”, your identity will remain anonymous but your review may still be made public.


For information about this choice, including consent withdrawal, please see our [Privacy Policy](https://www.plos.org/privacy-policy).

Reviewer #1: No

Reviewer #2: No

[NOTE: If reviewer comments were submitted as an attachment file, they will be attached to this email and accessible via the submission site. Please log into your account, locate the manuscript record, and check for the action link "View Attachments". If this link does not appear, there are no attachment files.]

While revising your submission, please upload your figure files to the Preflight Analysis and Conversion Engine (PACE) digital diagnostic tool, https://pacev2.apexcovantage.com/. PACE helps ensure that figures meet PLOS requirements. To use PACE, you must first register as a user. Registration is free. Then, login and navigate to the UPLOAD tab, where you will find detailed instructions on how to use the tool. If you encounter any issues or have any questions when using PACE, please email PLOS at figures@plos.org. Please note that Supporting Information files do not need this step.
