# Supplementary material for: The translation and validation of the Organ Transplant Symptom and Well-Being Instrument in China
Source: PLOS Glob Public Health. 2022 Sep 28;2(9):e0000718. doi: 10.1371/journal.pgph.0000718 (PMC10021454; doi:10.1371/journal.pgph.0000718)
Supplement: Attachment — Submitted filename: Response to Reviewers.docx [file pgph.0000718.s004.docx]

Dear Editors and Reviewers:

Thank you for your letter and for the reviewers’ comments concerning our manuscript entitled " The translation and validation of the Organ Transplant Symptom and Well-Being instrument in China " ( PGPH-D-21-00455). Those comments are all valuable and very helpful for revising and improving our paper, as well as the important guiding significance to our research. We have studied comments carefully and have made correction which we hope to meet with approval. Revised portions are marked in red in the paper. The main corrections in the paper and the responds to the reviewer’s comments are as flowing:

Editor Comments

1. Check the correct use of English in the manuscript, so the adequate use of terms like: farm, retire, man, woman
Thanks a lot. We revised this part. We change farm to farming,retire to retirementment, man to male, woman to female.

2. The test-retest reliability section should be corrected. You suggest to use one test, and finally ended up using other.
Thanks a lot. We revised this part. Change the article content to in all, 30 participants completed the retest questionnaire in order to assess the test-retest reliability over a two-week interval. A Spearman’s rank correlation coefficient (r) of 0.30 or lower is considered weak, between 0.30 and 0.60 is considered moderate, and ≥ 0.60 is considered strong(1). The intraclass correlation coefficients showed a high test-retest reliability (r = 0.713, p < .001) for the C-OTSWI.

3. References: 3, 13, 53, should be double-checked
Thanks a lot. We revised this part. We changed reference 3 to Tarabeih M, Bokek-Cohen Y, Azuri P. Health-related quality of life of transplant recipients: a comparison between lung, kidney, heart, and liver recipients. Qual Life Res. 2020;29(6):1631-9.

We changed reference 13 to Drevet G, Duruisseaux M, Maury JM, Riche B, Grima R, Ginoux M, et al. Lung cancer surgical treatment after solid organ transplantation: A single center 30-year experience. Lung cancer (Amsterdam, Netherlands). 2020;139:55-9.

We changed reference 53 to Shahabeddin Parizi A, Krabbe PFM, Buskens E, Bakker SJL, Vermeulen KM. A Scoping Review of Key Health Items in Self-Report Instruments Used Among Solid Organ Transplant Recipients. The patient. 2019;12(2):171-81.

4. Include texts in supplementary material, in both languages

Thanks a lot. We revised this part. We have added the Chinese version of the scale to the supplementary material.

Reviewer #1: All comments and suggestions are in the attached files. The quality of the study was evaluated applying the STROBE checklist and the recommendations by section were abandoned.


1. It is considered important that the authors include a paragraph on the control of bias in the study.

Thanks a lot. We revised this part. We realize that controlling for bias is a very important element of the study. In the text we have added this section “Our participants were all from The First Affiliated Hospital of China Medical University in Shenyang . The data were collected from November 2020 and January 2021 ”.

2. The study proposes a factorial analysis. Given that there is a hypothesis about the factorial structure of the instrument, it is suggested being explicit about that hypothesis and indicate that it is NOT an exploratory analysis, but a confirmatory one; and make explicit why it was decided to do a factorial analysis of principal components using a varimax rotation.
Finally, ¿why does the study use the Pearson correlation and not the Spearman correlation if the variables are ordinal? I suggest change that correlation or show that the variables meet the assumptions.

Thanks a lot. We revised this part. We added hypotheses and used factor analysis for validation in the article. KMO and Bartlett ball inspection were conducted to determine suitability for factor analysis using principal component analysis and maximum variance orthogonal rotation method to extract the characteristic value(2).

The analysis used in the article was conducted by Spearman, and it was our lack of rigor that led to the writing error. We subjected the data to a normality test and found that it was not normally distributed, so we used Spearman.

3. In the methodology the authors say that “A value of > 0.40 was considered to be substantial” and then in the results the authors say that “A correlation coefficient of lower than 0.3 was adopted as the basis for question deletion”Why they change the first assumption? A correlation of 0.3 is considered low, and a candidate for deletion.
The authors say “Correlations between individual items and the total score of the C-OTSWI were significant and positive for the entire sample.”But It’s not the same the statistical significance and the strength of association. Seven items have a correlation between individual items lower that 0.4 and two lower that 0.3; It’s important analyse is it’s better eliminate those items or explain very well why they don’t do that.In the methodology the authors say that they will use a Pearson correlation, but in the results, they write about a Spearman’s rank correlation coefficient and then report an intraclass correlation coefficients as a result. They must change that and be consistent between methodology and results.

Thanks a lot. We revised this part. Due to our lack of rigor resulting in inconsistent expressions in the article, our data are based on >0.3(1). We have checked the full text and corrected such errors. We are using spearman correlation and think that less than 0.3 is needed to delete, although the correlation coefficient between the item26 and 27 the overall were low in the scale, such problems were found in the communication with the patients, so the item26、27 were retained.

4. The authors include some limitations, but nothing about the lack of bias control
Thanks a lot. We revised this part and aware of this problem. We added the limitation of not being able to fully control for bias in the article.

5. The authors say: “The high correlation coefficient shows that the items of the scale have a strong relationship to the scale construct.”There was not high correlation in all cases, my recommendation is to be conservative in the discussion and more precise based on the results

Thanks a lot. We revised this part. In the discussion section of the article, we added a note about this section. The test showed that the Chinese version of the scale was usable, and although the correlation coefficient between the item26 and 27 the overall were low in the scale, such problems were found in the communication with the patients, so the item26、27 were retained. In future studies, we can conduct a multicenter survey to test this further.

Reviewer #2: The manuscript responds to a need in evaluating the outcomes and prognosis of transplant activity. The use of the OTSWI instrument has broadened the horizon of the evaluation of transplant patients beyond physical well-being and allows the senses of physical, mental and emotional well-being to be included. The methodology used both to adapt the scale from the point of view of the language and with the linguistic and cultural aspects of the conception of the disease, is absolutely appropriate. Regarding the methodology to evaluate the validity and internal consistency, reliability and concurrent validity, the approach, the results and the significance are interesting and clearly important and applicable.

Thanks a lot.

We tried our best to improve the manuscript and made some changes in the manuscript. These changes will not influence the content and framework of the paper. And here we did not list the changes but marked in red in revised paper.

We appreciate for Editor and Reviewers’ warm work earnestly, and hope that the correction will meet with approval.

Once again, thank you very much for your comments and suggestions.

**Reference**

1.	DeVon HA, Block ME, Moyle-Wright P, Ernst DM, Hayden SJ, Lazzara DJ, et al. A psychometric toolbox for testing validity and reliability. J Nurs Scholarsh. 2007;39(2):155-64.

2.	Liu YQ, Xiong SQ, Sang M, Li YF, Anarte Ortiz MT, Xing QL, et al. Reliability and validity of the Chinese version of the new fear of hypoglycemia scale: FH-15. International journal of nursing sciences. 2018;5(4):343-51.
